# Sanjad–Sakati Syndrome in Jordan: Clinical Features, Comorbidities, and Mortality Rate

**DOI:** 10.1055/s-0046-1822812

**Published:** 2026-05-11

**Authors:** Abdelrazaq Ahmad Alyasin, Fadi Ayyash, Hadeel Al Qurieny, Sondos Al Harahsheh, Asmaa Al Quraan, Waed Itewi, Saif Batarseh, Aseel Al Nemri

**Affiliations:** 1 Pediatric Endocrine and Diabetes Department, Queen Rania Al Abdullah Hospital for Children, King Hussein Medical Center, Amman, Jordan; 2 Pediatric Emergency Department, Queen Rania Al Abdullah Hospital for Children, King Hussein Medical Center, Amman, Jordan; 3 Pediatric Infectious Disease Department, Queen Rania Al Abdullah Hospital for Children, King Hussein Medical Center, Amman, Jordan

**Keywords:** Sanjad–Sakati syndrome, hypoparathyroidism, hypocalcemia, short stature

## Abstract

**Background:**

Sanjad–Sakati Syndrome (SSS) is a rare autosomal recessive disorder characterized by congenital hypoparathyroidism, dysmorphic features, and severe growth failure. This study aims to examine the clinical picture, comorbidities, and mortality rate of SSS in Jordan.

**Methods:**

This retrospective study included all patients diagnosed with SSS at the pediatric endocrinology clinics of Queen Rania Al Abdullah Hospital for Children, Amman, Jordan, between January 2002 and August 2025. Twenty-two children were evaluated for demographic characteristics, growth parameters, biochemical findings, comorbidities, and mortality rate.

**Results:**

Of the 22 patients (age range: 4 months–23 years), 14 were male and 8 were female. All patients presented with low birth weight, dysmorphic features, hypocalcemia, congenital hypoparathyroidism, and short stature. Parental consanguinity was recorded in 88.8% of the families. Nephrocalcinosis occurred in 31.8% of the patients ( *n* = 7), chronic intestinal pseudo-obstruction in 22.7% ( *n* = 5), and subclinical hypothyroidism in 9.1% ( *n* = 2). Ten patients (45.5%) died, most commonly due to pneumonia and sepsis.

**Conclusion:**

Patients with SSS in Jordan consistently presented with congenital hypoparathyroidism, developmental delay, intellectual disability, and severe growth failure. Frequent comorbidities include nephrocalcinosis, dental anomalies, intestinal pseudo-obstruction, and complications from chronic calcium therapy. These comorbidities are associated with increased mortality rate in SSS.

## Introduction

Sanjad–Sakati syndrome (SSS), also known as hypoparathyroidism–mental retardation–dysmorphism syndrome (HRD), is a rare autosomal recessive inherited disorder. It was first reported in 1988 and confirmed in 1991 as a syndrome of congenital hypoparathyroidism associated with small gestational age and dysmorphism. 1 The condition has been reported predominantly in families hailing from the Arabian Peninsula, including Saudi Arabia, Kuwait, Jordan, Egypt, Oman, and Sudan. Affected individuals typically present with a long narrow face, micrognathia, beaked nose, deep-set small eyes, large floppy ears, severe pre- and post-natal growth failure, and intellectual disability. 2 Newborns often present with seizures or apnea due to hypocalcemia. 3

SSS is caused by mutations in the tubulin-folding cofactor E (TBCE) gene located on chromosome 1q42-43. This gene encodes one of the five chaperone proteins essential for proper α-tubulin folding and the formation of α–β-tubulin heterodimers. 4 TBCE is also believed to play a critical role in parathyroid gland development. 5

The diagnosis of SSS is established through a combination of medical history (family history of SSS, gestational age, and birth weight), clinical features (dysmorphic features such as deep-set small eyes, micrognathia, narrow face, large floppy ears, and short stature), laboratory findings (hypoparathyroidism, hypocalcemia), and genetic studies (mutations in the TBCE gene on chromosome 1q42-43).

Short stature is one of the most common findings in SSS, with a prevalence rate of 100%. Although the underlying cause of this condition is unclear, a primary defect related to TBCE mutation is suspected. 6 While some patients show a normal growth hormone (GH)–insulin-like growth factor-1 axis and are categorized as having idiopathic short stature or being small for gestational age without catch-up growth, others may exhibit GH resistance. The efficacy of GH therapy in these patients remains uncertain and controversial. 7

Complications of chronic calcium replacement therapy include soft tissue calcifications (affecting the brain, eyes, teeth, and kidneys) and chronic constipation. Studies have recommended routine thyroid function and antibody screening in SSS patients due to the risk of autoimmune thyroiditis, which can impair growth and neurodevelopment. 8 Chronic intestinal pseudo-obstruction has also been reported, likely due to congenital, permanent hypoparathyroidism, and hypocalcemia. Symptoms include intermittent obstruction often relieved with conservative management, although surgical intervention may be necessary in some cases. 9

Treatment of SSS primarily focuses on managing neonatal hypocalcemia with calcium and active vitamin D (1-alpha hydroxycholecalciferol). Given the frequent association with low birth weight, adequate nutritional support is essential. Although data are limited and syndrome-specific growth charts are unavailable, GH therapy may be considered in some cases, particularly in children born small for gestational age who fail to exhibit catch-up growth after 2 to 4 years. Ongoing follow-up with dental, nephrology, gastroenterology, and neurology specialists is critical for comprehensive management.

This study aims to describe the clinical characteristics, complications, and outcomes of children with genetically confirmed SSS in Jordan, highlighting local experiences and management challenges.

## Materials and Methods

### Study Data Selection and Variables

This retrospective study included all children diagnosed with SSS between January 2002 and August 2025 at the pediatric endocrinology clinics in Queen Rania Al Abdullah Hospital for Children, Amman, Jordan.

Twenty-two patients presented with hypocalcemia, dysmorphic features, and low birth weight. All patients underwent serial tests, including full blood count, calcium, magnesium, phosphate, alkaline phosphatase, albumin, creatinine, 24-hour urinary (phosphate, calcium, magnesium, and creatinine), parathyroid hormone, thyroid-stimulating hormone, free thyroxine (T4), thyroglobulin antibodies (Tg Ab), thyroid peroxidase antibodies (TPO), vitamin D, chest X-ray, renal ultrasound, and echocardiograms. In addition, fluorescence in situ hybridization was performed in all patients to rule out DiGeorge syndrome.

### Genetic Testing

Seventeen patients underwent direct mutation analysis targeting the 12 base pair (bp) deletion (155–166 del) in exon 3 of the TBCE gene, conducted using polymerase chain reaction (PCR) for genetic testing to identify TBCE mutations. All tested patients were found to carry this deletion. The remaining five patients were diagnosed clinically based on classical phenotype and family history.

### Treatment and Follow-Up

All patients received calcium replacement therapy (initially with calcium gluconate and then calcium carbonate) and active vitamin D (1-alpha hydroxycholecalciferol). Monitoring was performed through frequent laboratory evaluations during dose adjustments. All patients were followed up weekly to monthly, and then quarterly or biannually to assess treatment effectiveness and detect complications such as hypercalciuria, nephrocalcinosis, constipation, and renal impairment.

### Statistical Analysis

Statistical analysis was performed using SPSS (v26; IBM Corporation, Armonk, New York, United States). Continuous variables were expressed as mean ± standard deviation (SD), while categorical variables were expressed as frequencies and percentages.

### Ethical Statement

Ethics approval was obtained from the Jordanian Royal Medical Services, and informed consent was obtained from the patients' parents.

## Results

**Fig. 1:**
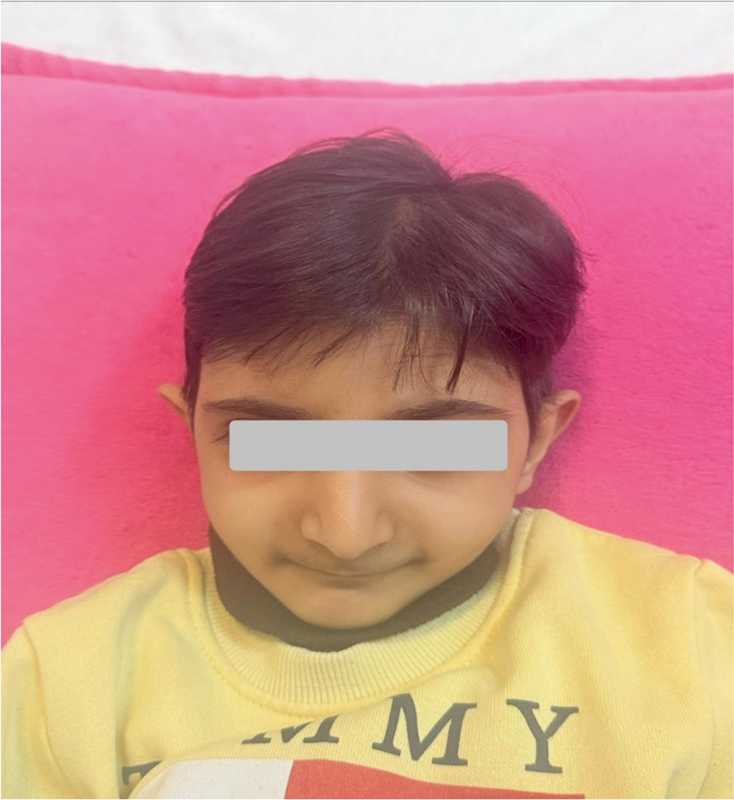
One of our patients.

This study examined 22 patients diagnosed with SSS from 18 Jordanian families between January 2002 and August 2025. Their age range was from 4 months to 23 years, with a mean age of 10.17 years (SD = 5.31) and a median age of 9.5 years. Of the total patients, 63.6% were male ( *n* *=* 14) and 36.4% were female ( *n* = 8). Twenty patients were born at full term via normal vaginal delivery, while two patients were born prematurely via cesarean section. Parental consanguinity was reported in 88.8% of the families ( *n* = 16). All patients exhibited characteristic dysmorphic features, as shown in Table 1 and Fig. 1 . In addition, all children ( *n* = 22) were diagnosed with low birth weight (< 2,500 g), hypoparathyroidism, and hypocalcemia. Dental abnormalities including enamel hypoplasia, microdontia, and severely carious teeth were present in 90.9% of the patients ( *n* *=* 20). Short stature was likewise observed in 90.9% of the patients ( *n* = 20).

**Table 1:** Dysmorphic features of Sanjad–Sakati syndrome

Feature	Percentage
IUGR	100
Hypoparathyroidism	100
Microcephaly	100
Small hands and feet	100
Deep-set eyes	100
Short stature	100
Intellectual disabilities	100
Beaked nose	100
Depressed nasal bridge	100
Consanguinity	88.8
Large floppy earlobes	63.6
Teeth anomalies	90.9

Abbreviation: IUGR, intrauterine growth restriction.

Renal nephrocalcinosis developed in 31.8% of the patients ( *n* = 7) as a complication of continuous calcium replacement therapy followed by pediatric nephrology. Subclinical hypothyroidism with negative thyroid antibodies (TPO and Tg Ab) was observed in 9.1% of the patients ( *n* = 2). Recurrent episodes of abdominal distension, bilious vomiting, and constipation occurred in 22.7% of the patients ( *n* = 5). Plain abdominal radiographs and contrast barium swallow showed dilatation from the duodenum to the rectum without an identifiable structural cause of obstruction. In most cases, remission of abdominal symptoms was achieved with conservative management; however, one of the five patients failed to improve and required laparotomy, after which he died postoperatively due to septic shock.

All patients ( *n* = 22) were treated with calcium supplements and an active form of vitamin D. Overall, 45.5% of the patients ( *n* *=* 10) died due to pneumonia and sepsis. Table 2 shows the most common complications associated with SSS.

**Table 2:** Complications of patients with Sanjad–Sakati syndrome

Complication	Number of patients ( *n* )	Percentage (%)
Nephrocalcinosis	7	31.8
Subclinical hypothyroidism	2	9.1
Chronic intestinal pseudo-obstruction	5	22.7
Pneumonia/sepsis-related deaths	10	45.5
Dental problems	20	90.9

## Discussion

SSS is a rare autosomal recessive disorder. Its incidence in Kuwait is estimated at 7 to 18 per 100,000 live births, 10 while the estimated incidence in Saudi Arabia varies from 1:40,000 to 1:100,000 live births. 11 No incidence data are currently available for Jordan.

Despite a significant decline in consanguinity rates in Amman, Jordan, in recent generations, 12 the consanguinity rate among families with SSS remains significantly high at 88.8%, approximately similar to the findings of Aminzadeh et al, 4 and much higher than the 12.5% reported by Naguib et al. 10

Consistent with previous reports, 13 14 this study also recorded significant prenatal and postnatal growth impairment. Twenty patients were born at full term via normal vaginal delivery, while two patients were born prematurely via cesarean section. All children in our study were diagnosed with low birth weight.

This study confirmed the triad of congenital hypoparathyroidism, growth failure, and intellectual disabilities as defining features of SSS. The most common dysmorphic features in our patients were microcephaly, deep-set eyes, depressed nasal bridge, micrognathia, small hands and feet, and short stature. 15 16 17

In our study, 17 patients underwent genetic testing using PCR to confirm TBCE mutations. Each of these patients demonstrated a 12 bp (155–166 del) deletion in exon 3 of the TBCE gene. Notably, all reported cases from the Middle East to date have shown this deletion. 9 18

SSS should be differentiated from Kenny–Caffey syndrome, which shares overlapping features with SSS but is distinguished by normal intelligence, bone abnormalities (small, slender, long bones with medullary stenosis and thickened cortex), and macrocephaly ( Table 3 ). 10 19 20

**Table 3:** Differentiation between Sanjad–Sakati syndrome and Kenny–Caffey syndrome, type 1 and 2

Feature	Sanjad–Sakati syndrome	Kenny–Caffey syndrome type 1	Kenny–Caffey syndrome type 2
Craniofacial	Microcephaly Micrognathia Deep-set eyes Long philtrum Posteriorly rotated ears	Microcephaly Broad cheeks Hypertelorism Dental caries	Macrocephaly Nanophthalmos Cornea and retina calcification Congenital cataract
Skeletal	Delayed bone age Small hands and feet Patchy osteosclerosis	Delayed bone age Poorly ossified skull bones Calvarial osteosclerosis Medullary stenosis of tubular bones Small hands and feet	Osteosclerosis Thickened cortex and narrow marrow cavities of long bones
Mental	Intellectual disability (mild–moderate)	Intellectual disability/Normal mental health	Normal mental health
Other	Micropenis Cryptorchidism Ventricular dilatation	–	–
Laboratory findings	Hypocalcemia Low parathyroid hormone Hypophosphatemia Normal cell-mediated immunity	Hypocalcemia Low parathyroid hormone Low to low–normal magnesium	Transient hypocalcemia Low parathyroid hormone Transient hypophosphatemia Deficient T-cell function
Molecular pathology	Mutation in tubulin-specific cofactor E gene	Mutation in tubulin-specific cofactor E gene	Unknown
Inheritance	Autosomal recessive	Autosomal recessive	Autosomal dominant/X-linked

Adapted from: Naguib KK, Gouda SA, Elshafey A, Mohammed F, Bastaki L, Azab AS, et al. Sanjad-Sakati syndrome/Kenny-Caffey syndrome, type 1: a study of 21 cases in Kuwait, East Mediterr Health J [online]. 2009; 15(2):345-352 [Accessed December 9, 2025]. Available from: https://pubmed.ncbi.nlm.nih.gov/19554981/ .

Consequently, distinguishing between the two syndromes should rely on a comprehensive evaluation of the clinical presentation, supported by biochemical, radiological, and, if feasible, molecular diagnostics.

All patients in our study (100%, *n* = 22) exhibited short stature. However, due to limited data, the high cost of GH therapy, poor compliance, and parental refusal in some cases for GH stimulation testing, we were unable to assess the effect of GH treatment in SSS. Al Jurayyan et al reported that 83.3% of patients were not GH deficient, 21 whereas Rafique and Al-Yaarubi suggested that the primary issue in SSS is GH resistance. 7

Oral manifestations, including enamel hypoplasia, microdontia, and severely carious teeth, were observed in 90.9% of the patients. These features are thought to result from the hypoparathyroidism and hypocalcemia associated with this syndrome. Similar findings have been reported by Hassona et al 11 and Al-Malik. 19

We also found that 31.8% of SSS patients developed nephrocalcinosis (calcium salts deposited in the kidney), which is often associated with congenital hypoparathyroidism due to an imbalance between calcium and phosphorus (high phosphorus and low calcium). Moreover, treatment with calcium and calcitriol (the active form of vitamin D) also increases the risk of nephrocalcinosis. Previous case reports have documented this complication, with Laimon et al 22 reporting bilateral medullary nephrocalcinosis in 59% of patients.

Chronic intestinal pseudo-obstruction was observed in 22.7% of the patients ( *n* = 5), presenting with recurrent episodes of abdominal distension, bilious vomiting, and constipation. Several previous case reports have highlighted a strong association between SSS and recurrent intestinal pseudo-obstruction, primarily attributed to hypocalcemia-induced smooth muscle dysfunction. 23 24

In our study, two patients (9.09%) were diagnosed with subclinical hypothyroidism accompanied by negative thyroid antibodies (TPO and Tg Ab). This association has been previously recognized by Anteet et al, 8 and further studies are warranted to explore the association between SSS and Hashimoto's thyroiditis.

Mortality was notably high, with 45.5% of the patients ( *n* = 10) dying, most of them during childhood (ages 4–12 years), primarily from pneumonia and sepsis, similar to other reports, 25 26 which found no immunological defects. A descriptive comparison between deceased and surviving patients suggests that mortality may be linked to a higher burden of systemic complications. Patients who died more frequently experienced recurrent infections, severe gastrointestinal involvement such as chronic intestinal pseudo-obstruction, and complications requiring surgical intervention. In addition, prolonged disease course and cumulative effects of metabolic instability may have contributed to poorer outcomes. However, due to the limited sample size and lack of inferential statistical analysis, these observations should be interpreted with caution and cannot be considered predictive. Future studies with larger cohorts are required to better identify risk factors associated with mortality in this population.

### Study Limitations

This study has several limitations. First, the sample size is relatively small, reflecting the rarity of SSS, which limits the ability to perform robust statistical analyses or identify independent predictors of mortality. Second, genetic confirmation was available for only 17 out of 22 patients, while the remaining cases were diagnosed based on clinical features and family history, which may introduce diagnostic heterogeneity. Third, the study was conducted at a single tertiary center, which may limit the generalizability of the findings to other populations. Finally, the lack of a control or comparison group restricts the ability to draw causal inferences regarding factors associated with mortality.

## Conclusion

This study provides a comprehensive description of the clinical features, comorbidities, and outcomes of children with SSS in Jordan. The condition is consistently characterized by congenital hypoparathyroidism, hypocalcemia, severe growth failure, and intellectual disability, with a high burden of associated complications, particularly nephrocalcinosis, dental abnormalities, and chronic intestinal pseudo-obstruction.

A notably high mortality rate was observed, predominantly due to infections such as pneumonia and sepsis. Early diagnosis, close monitoring, and comprehensive care can help improve the quality of life and reduce morbidity and mortality in affected individuals. Further research is warranted to clarify the long-term clinical outcomes of patients with SSS.
